# Factors associated with short-term recurrent ischemic stroke: culprit plaque, collateral circulation, and pathological mechanisms

**DOI:** 10.3389/fneur.2025.1720513

**Published:** 2025-12-18

**Authors:** Gen Li, Di-hao Xu, Min Shu, Qian-kun Chu, Zhi-bin Zeng, Li-ping Ma

**Affiliations:** Shenzhen Nanshan People’s Hospital, Shenzhen, China

**Keywords:** high-resolution vessel wall imaging, CTP, border zone infarcts, ischemic stroke recurrence, intracranial atherosclerotic disease

## Abstract

**Purpose:**

The purpose of the study was to investigate the predictive value of baseline plaque characteristics, perfusion injury, and pathological mechanisms for new ischemic cerebral lesions (NICLs) in medically treated patients with acute ischemic stroke (AIS) due to intracranial atherosclerotic stenosis (sICAS).

**Methods:**

This was a retrospective analysis of AIS patients with 50–99% middle cerebral artery M1 segment (MCA-M1) stenosis undergoing high-resolution vessel wall magnetic resonance imaging (HR-VWI) and computed tomography perfusion (CTP) within 1 week of admission to evaluate plaque characteristics and collateral status (CS). sICAS pathogenesis was classified into three subtypes: artery-to-artery embolism (A-A), border zone infarction (BZI), and penetrating artery occlusion, primarily based on infarct topography. Baseline clinical/imaging features and pathogenic mechanisms were compared between patients with and without NICLs during 1-year follow-up. A Cox proportional hazards regression model was used to identify independent risk factors associated with stroke recurrence.

**Results:**

Among the 78 eligible patients, 25 developed NICLs, showing significantly higher adverse CS (60.0% vs. 24.5%, *p* = 0.005) and a larger mismatch area (*p* = 0.043) than the non-NICL group, with no plaque feature differences. NICL’s incidence varied significantly among pathogenesis subtypes (*p* = 0.003), with baseline BZI demonstrating higher recurrence rates than A-A (56% vs. 28%, *p* = 0.001). Multivariable Cox regression analysis found that BZI [adjusted hazard ratio, 3.28 (95% CI, 1.49–7.36); *p* = 0.004] and unfavorable CS [adjusted hazard ratio, 2.87 (95% CI, 1.16–7.09); *p* = 0.011] were independently associated with the NICLs.

**Conclusion:**

In the risk stratification for short-term recurrence for medically managed AIS, baseline CT perfusion deficits are prioritized over plaque imaging. Moreover, unfavorable CS and the pathogenic mechanism of BZI indicate compromised hemodynamics and serve as the pivotal and independent predictors of early relapse.

## Introduction

The risk of new ischemic cerebral lesions (NICLs) in acute ischemic stroke (AIS) patients due to intracranial atherosclerotic stenosis (sICAS) was significantly reduced after antiplatelet combined with stringent vascular risk factor management (1, 2). However, some patients experienced relapse, suggesting that the current treatment strategy may not fully address the underlying pathophysiological mechanisms in all individuals. Therefore, the identification of patients at high risk for recurrence through precise baseline imaging has become a critical issue in clinical management.

In recent years, high-resolution vascular wall imaging (HR-VWI), a non-invasive *in vivo* imaging technique capable of assessing arterial wall morphology with high spatial resolution, has emerged as a valuable tool for identifying high-risk atherosclerotic plaques in AIS (3, 4). Thus, HR-VWI holds promise as a practical modality for patient-specific risk stratification.

For AIS patients caused by sICAS, the ongoing thromboembolism causes an increase in the degree of stenosis, reduced cerebral perfusion, or occlusion of branches originating from the affected artery. Alternatively, plaque rupture may result in partial or complete detachment, leading to distal embolization. Based on the underlying pathophysiology, sICAS-related AIS can be classified into four subtypes: border zone infarction (BZI), parent artery occlusion of penetrating arteries (POPAs), artery-to-artery embolism (A-A), and mixed mechanisms.

Recent HR-VWI studies (5, 6) have revealed that culprit plaque characteristics differ among these mechanisms. For instance, intraplaque hemorrhage (IPH) and irregular plaque surfaces—hallmarks of vulnerable plaques—were more commonly associated with A-A infarction. The incidence of positive remodeling in culprit plaques was more prevalent in the POPA subtype than in other types of stroke mechanisms. Additionally, the enhancement ratio in the POPA group had been found to be lower than that in other mechanism groups (5). These findings suggested that proximal thromboembolic sources with different pathophysiological mechanisms exhibit distinct plaque characteristics, potentially leading to varying risks of stroke recurrence. Consequently, differences in disease progression and clinical outcomes may exist among these subtypes.

Moreover, computed tomography perfusion (CTP) offers a rapid and cost-effective means of evaluating collateral status (CS) (7) and can precisely delineate between irreversibly injured and potentially salvageable tissue, which is crucial for guiding treatment decisions. The relative cerebral blood flow (CBF) volume is regarded as an indicator of the degree of cerebral blood flow compensation and serves as a reliable perfusion-based parameter for evaluating CS (7, 8). Additionally, cerebral blood volume (CBV) represents blood flow reserve capacity for tissue sustenance.

Therefore, we hypothesize that embolism arising from unstable plaques and CS may exert independent effects or interact in a complementary manner, thereby inducing ischemic recurrence in sICAS-associated AIS patients. In this retrospective study, our purposes were: (1) to demonstrate the correlation between baseline culprit plaque features/perfusion deficits and the development of NICLs and (2) to identify subgroups of mechanisms at high risk of recurrence that may benefit from more tailored therapies targeting mechanisms to prevent stroke recurrence.

## Materials and methods

**Figure 1:**
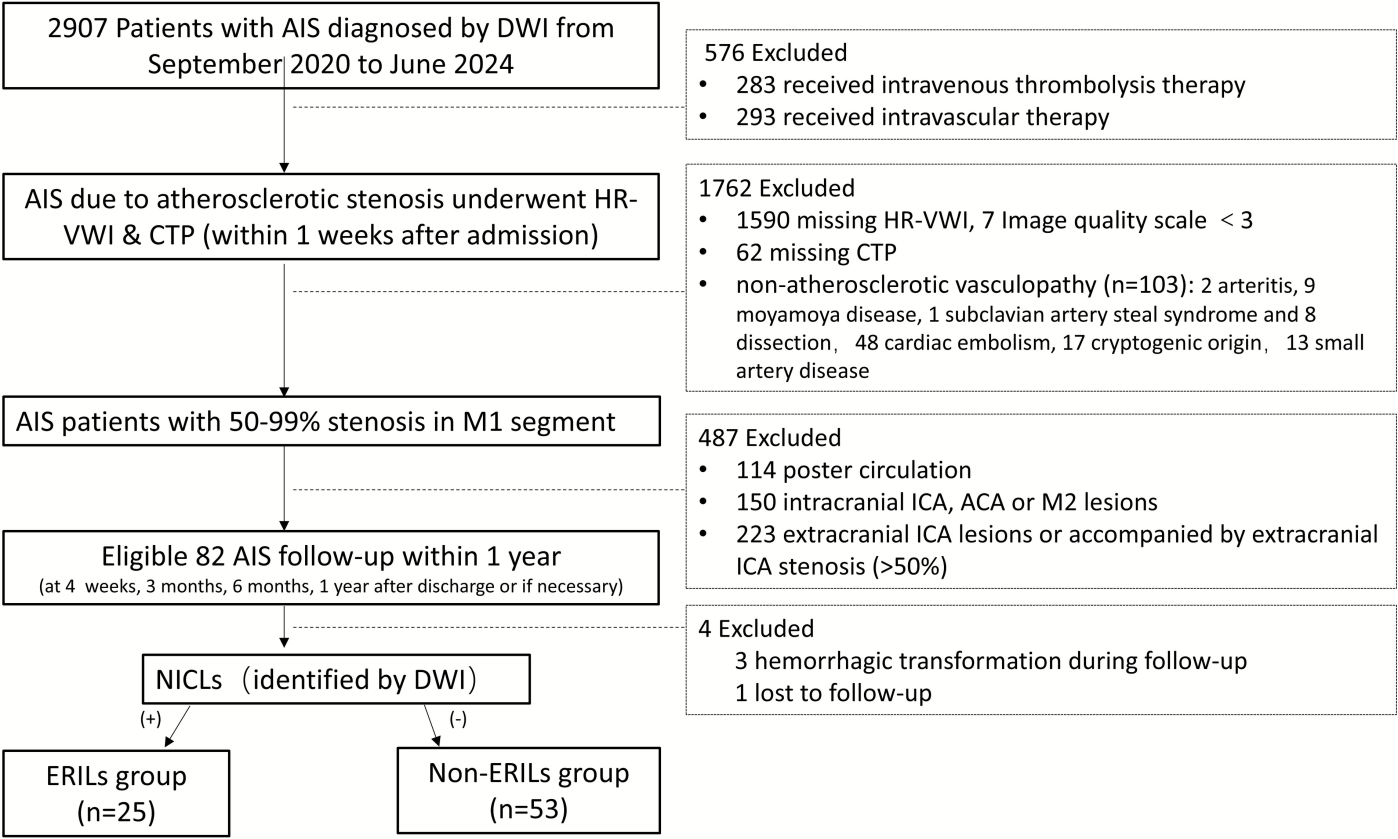
Flowchart. AIS, acute ischemic stroke; DWI, diffusion-weighted imaging; HRVWI, high-resolution vessel wall imaging; CTP, computed tomography perfusion; NICLs, new ischemic cerebral lesions; ERILs, early recurrence ischemic lesions.

This study was a retrospective, short-term longitudinal observational design and had been approved by the ethics committee of our hospital. Given its retrospective nature and the use of only de-identified and anonymized patient data, the ethics committee waived written informed consent. From September 2020 to June 2024, patients with AIS diagnosed by diffusion-weighted imaging (DWI) were evaluated. Those who received intracranial HR-VWI and CTP within 7 days after admission were included in this study. The inclusion criteria were as follows: (1) AIS due to large artery atherosclerosis, as defined by the Trial of Org 10,172 in Acute Stroke Treatment, was confirmed by diffusion-weighted imaging (DWI) within 7 days of symptom onset; (2) intracranial HR-VWI and CTP examinations were performed within 1 week of admission; (3) the culprit plaque was located in the M1 segment of the MCA, with severe stenosis (greater than 50%); and (4) patients did not receive reperfusion therapy (intravenous thrombolysis or intravascular therapy) after admission. The exclusion criteria included: (1) vascular diseases of other etiology, such as arteritis, moyamoya disease, subclavian artery steal syndrome, and dissection; (2) evidence of suspected cardiac embolism (atrial fibrillation, mitral stenosis or artificial valves, sick sinus syndrome, and acute bacterial endocarditis), cryptogenic origin, or small artery disease; (3) patients with transient ischemic attack (TIA) or chronic stroke without high-intensity lesions on DWI, as these could not be classified according to the pathological mechanism; (4) presence of significant extracranial arterial stenosis (>50%); (5) incomplete clinical data or inadequate HR-VWI and CTP imaging; (6) image quality does not meet the measurement standards due to motion or flow artifacts; and (7) hemorrhagic stroke occurred during the follow-up period. A flowchart of the included and excluded criteria is presented in Figure 1 in detail.

Baseline demographic and clinical information, including age, gender, hypertension, diabetes, dyslipidemia, hyperhomocysteinemia, body mass index (BMI), and smoking, as well as the National Institutes of Health Stroke Scale (NIHSS), was reported in the medical record system and collected by neurologists.

### Standard medical treatment

All patients admitted with AIS at our institution were managed according to the contemporary national and international stroke prevention guidelines (9, 10). The routine best medical treatment typically included, but was not limited to, antiplatelet therapy (e.g., aspirin or clopidogrel), high-intensity statins, and strict control of vascular risk factors such as hypertension and diabetes. Data regarding the medications administered during hospitalization and prescribed at discharge were retrieved and verified by reviewing electronic medical records by the physicians (MS and GL).

### Study outcome measures

Patients were followed up and evaluated in the neurology or rehabilitation outpatient department at 4 weeks, 3 months, 6 months, and 1 year after discharge or if necessary. Patients with a suspected stroke recurrence underwent brain MRI to assess for NICLs. For patients without outpatient follow-up, telephone follow-up was performed by the physicians (MS and GL). To clarify the relationship between recurrent lesions and culprit arterial supply, the primary endpoint was NICLs in the ipsilateral territory of anterior circulation during a 1-year follow-up period; an enlargement of the primary lesion within 21 days was not considered an NICL. NICLs were further classified into different pathogeneses based on the territory.

### Neuroimaging acquisition

Three-dimensional (3D) high-resolution vessel wall imaging (HR-VWI) was performed using a 3.0 Tesla MRI system (SIGNA Pioneer, GE Healthcare). T1-weighted, T2-weighted, and contrast-enhanced T1-weighted images were acquired using double inversion recovery black-blood sequence. Protocol details are provided in Supplementary Table 1. Initial and follow-up brain MRIs, including magnetic resonance angiography (MRA), and routine CTP were also acquired.

### Baseline images post-process and analysis

The image quality of HR-VWI was scored as 4 points based on the signal-to-noise ratio, where 1 means the lowest quality and 4 represents the highest quality. Images with scores ≥3 were considered capable of providing a clear visualization of the vessel wall. HR-VWI image processing was performed using the MR TWIST toolkit on the Syngo.via workstation (SIEMENS Healthcare). The semi-automatic extraction process commences with delineating the start and end points of the culprit plaque, which encompasses the proximal and distal reference segments, followed by manually refining the centerline. Multi-planar and curved-planar reconstructions were then generated automatically. In case of multiple lesions (two or more) in the ipsilateral M1 segment, the criminal was manifested as the most narrowed or the most markedly enhanced lesion. The reference site was set as the proximal normal lumen of the lesion. In cases where the lesion was diffuse, measurements from both the proximal and distal reference sites were recorded and averaged.

For CTP images, cerebral blood flow (CBF), cerebral blood volume (CBV), and tissue with delayed time to maximum (Tmax) maps were automatically processed by the StrokePortal workflow on the Digital Brain workstation (SK-CTPDoc, StrokePro V2.4, Shukun Technology, Beijing, China).

All VWI and CTP image analyses and measurements were carried out by three experienced radiologists (L-pM, D-hX, and Q-kC, who have 11 years, 8 years, and 26 years of diagnostic experience, respectively) who were blinded to clinical outcomes. Any discrepancies were resolved through discussion with and adjudication by a senior radiologist (Z-bZ), who has 32 years of extensive experience in cerebral vascular disease diagnosis.

### Assessment of plaque characteristics

A total of five key plaque characteristics associated with the recent ischemic event were included in the primary analyses: (1) presence of intraplaque hemorrhage (IPH), (2) the degree of enhancement, (3) plaque remodeling, (4) plaque length, and (5) multiple stenosis manifested by other intracranial vascular severe stenosis (greater than 50%). A detailed assessment and measurement of plaque characteristics was performed according to the practice guideline published by the American Society of Neuroradiology (11).

### Assessment of perfusion

CTP-based CS assessment was performed, as described in previous studies (12). Unfavorable CS was defined as a decrease in CBF, which could be accompanied by a further reduction in CBV. In contrast, favorable CS is characterized by preserved CBF when distal cerebrovascular resistance decreases through compensatory vasodilation, resulting in maintenance or elevation of CBV. CTP estimated ischemic core as relative CBF less than 30% of normal CBF. Tmax >6 s was used to identify hypoperfused tissue. The mismatch volume—representing the estimated the penumbral volume or tissue at risk—was calculated as the volume of critically hypoperfused tissue minus the volume of the ischemic core. Hypoperfusion mismatch volume was stratified by a cutoff value of mismatch volume (<10 mL) according to a previous study (13).

### Categorization of pathogenesis mechanisms

Ischemic stroke was classified according to pathogenesis, with prespecified criteria primarily on the basis of infarct topography and MRA, and independently assessed by two experienced physicians, who were blinded to outcomes. The detailed classification criteria were as follows:

#### POPA group

MCA stenosis ≥50% is located near the perforating artery ostium on MRA or VWI, and infarcts are located within the perforating artery range on DWI. These infarcts typically occur in the deep white matter or basal ganglia and may extend into the cortical regions.

#### BZI group

BZI primarily develops at the anatomical watershed zone between terminal branches of two major cerebral arteries, constituting a hemodynamically vulnerable region. BZI can be classified into two subtypes: external (cortical) BZI—typically manifests as wedge-shaped or ovoid lesions in regions supplied by the anterior and middle cerebral arteries (e.g., anterior external border zones and paramedian white matter) or the middle and posterior cerebral arteries (e.g., parieto-occipital areas)—and internal (subcortical) BZI—characterized by multiple, rosary-like lesions arranged in a linear pattern along the lateral ventricles, particularly within the centrum semiovale or corona radiata. Cases with predominantly BZI-related infarction, including mixed-type presentations, were also categorized under this classification.

#### A-A group

An A-A infarction is defined as ischemic lesions strictly confined to the vascular territory of the affected intracranial artery, encompassing both cortical and subcortical regions perfused by the diseased vessel, while explicitly excluding border zone areas.

### Statistical analysis

Statistical analyses were performed using IBM SPSS Statistics (version 22.0; IBM Corp.) and GraphPad Prism (GraphPad Software Inc.). Descriptive statistics included median with interquartile range (IQR) for continuous variables and frequencies with percentages for categorical variables. Intergroup comparisons of baseline characteristics were conducted using non-parametric Mann–Whitney *U* tests for continuous measures and Fisher’s exact tests for categorical variables, as appropriate. The chi-squared test was used to assess differences in recurrence rates among the three etiological subgroups. Furthermore, Cox proportional hazards regression analyses were performed to identify univariate and multivariate clinical predictors of NICLs. Hazard ratios (HR) with corresponding 95% confidence intervals (CI) were reported. A significance threshold of *p*-value <0.05 was applied for all statistical tests.

## Results

### Demographics

A total of 78 AIS patients (56.14 ± 1.45 years; 61 male) who received both VWI and CTP were recruited in this study with 50–99% stenosis in M1 segment lesions. Among the 78 AIS patients who received medical treatment alone, 40 had A-A infarcts, 26 had BZI, and 12 had POPA infarcts at baseline. During the 1-year follow-up, 25 (32%) patients were diagnosed with NICLs in the same arterial blood supply territory and were divided into the early recurrence ischemic lesion (ERIL) group, of which 14 (56%) patients had recurrent stroke within the first 3 months, and 3 of these patients underwent endovascular stenting after recurrence. The remaining 53 patients achieved functional recovery without the NICLs and were assigned to the non-ERIL group. Baseline characteristics of these patients are listed in Table 1. No significant differences were observed between the two groups in terms of clinical parameters. Although the baseline NIHSS score was higher in the ERIL group than in the non-ERIL cohort, the difference did not achieve statistical significance (*p* = 0.054).

**Table 1:** Comparison of baseline clinical, imaging features, and pathological mechanisms of AIS patients between the ERIL group and the non-ERIL group.

Parameters	All patient (*N* = 78)	ERILs group (*N* = 25)	Non-ERILs group (*N* = 53)	*p*
Demographics				
Age (y)	56.14 ± 1.45	59.06 ± 12.30	57.04 ± 11.03	0.246
Sex (male %)	61 (78.2)	18 (72.0)	45 (85)	0.222
Hypertension, *n* (%)	56 (70.59)	20 (77.27)	36 (67.39)	0.300
Diabetes, *n* (%)	29 (37.17)	7 (28.00)	21 (36.62)	0.449
TC (mmol/L)	4.53 (3.79, 5.34)	4.42 (3.61, 4.68)	4.64 (3.85, 5.52)	0.585
TG (mmol/L)	1.35 (1.048, 1.87)	1.60 (1.13, 2.15)	1.21 (1.03, 1.76)	0.260
HDL (mmol/L)	0.85 (0.77, 1.10)	0.83 (0.75, 0.93)	0.91 (0.77, 1.15)	0.548
LDL (mmol/L)	3.03 (2.17, 3.48)	2.77 (2.07, 3.11)	3.03 (2.17, 3.48)	0.220
Homocysteine (mmol/L)	11.55 (9.78, 13.98)	10.80 (9.65, 13.69)	12.00 (9.95, 14.13)	0.374
Smoking^a^	74 (29.7)	7 (31.8)	29 (55.8)	0.077
MBI^b^	25.70 (22.70, 27.50)	26.10 (23.10, 29.70)	25.45 (22.65, 27.18)	0.674
Initial NIHSS score	2 (0, 3)	2 (1, 6)	1 (0, 2)	0.054
Culprit plaque characteristics				
Remodeling pattern (% positive remodeling)	25 (32.1)	6 (24.0)	19 (35.8)	0.436
Plaque length (mm)	8.50 (6.0, 12.63)	7.15 (5.30, 13.33)	8.70 (6.40, 12.83)	0.333
Stenosis (%)	0.96 (0.92, 0.98)	0.97 (0.94, 0.98)	0.96 (0.91, 0.98)	0.151
IPH, *n* (%)	71 (91)	22 (88)	49 (92)	0.674
Multi-stenosis, *n* (%)	33 (42.31)	12 (48)	21 (39.62)	0.624
Enhancement grading, *n* (%)				0.118
Grade 0	5 (6.4)	0 (0)	4 (5.1)	
Grade 1	31 (39.7)	8 (32.0)	23 (43.4)	
Grade 2	42 (53.8)	17 (68.0)	25 (47.2)	
Perfusion parameters				
Unfavorable CS, *n* (%)	28 (35.9)	15 (60.0)	13 (24.5)	0.005^**^
Ischemic core volume, (mL)	0 (0, 0.5)	0 (0, 1.925)	0 (0, 0.15)	0.761
Penumbral volume, (mL)	8.6 (0, 48.93)	33.7 (0.35, 86.45)	5.1 (0, 33.9)	0.043^*^
Baseline pathological mechanisms, *n* (%)				0.008^**^
A-A	40 (51.3)	7 (28)	33 (62.3)	
BZI	26 (33.3)	14 (56.0)	12 (22.6)	
POPA	12 (15.4)	4 (16.0)	8 (15.1)	

AIS, acute ischemic stroke; ERILs, early recurrence ischemic lesions; TC, total cholesterol; TG, total triglycerides; HDL, high-density lipoprotein; LDL, low-density lipoprotein; BMI, body mass index; NIHSS, National Institutes of Health Stroke Scale; IPH, intra-plaque hemorrhage; CS, collateral status; A-A, artery-to-artery embolism; BZI, border zone infarcts; POPA, parent artery atherosclerosis occluding penetrating artery; ^*^p < 0.05; ^**^p < 0.01; ^a^*n* = 4 missing; ^b^*n* = 4 missing.

### Comparison of baseline plaque characteristics and perfusion parameters between the ERIL group and the non-ERIL group

No significant difference was detected in baseline plaque enhancement grade, IPH, remodeling patterns, or plaque length between the two groups, and the effect of positive remodeling on recurrence also had no significant difference (24.0% vs. 35.8%, *p* = 0.436). Although baseline unfavorable CS was significantly higher in patients of the ERIL group than in those of the non-ERIL group (60.0% vs. 24.5%, *p* = 0.005), with a larger mismatch area (*p* = 0.043), there was no difference in ischemic core volume on baseline CTP imaging (*p* = 0.761).

### Distribution of ischemic stroke mechanisms at baseline and 1-year follow-up

At baseline evaluation, the cohort of 78 acute ischemic stroke (AIS) patients demonstrated the following mechanistic distribution: 51.3% (40/78) exhibited A-A infarcts, 33.3% (26/78) exhibited BZI, and 15.4% (12/78) exhibited POPA infarcts.

**Figure 2:**
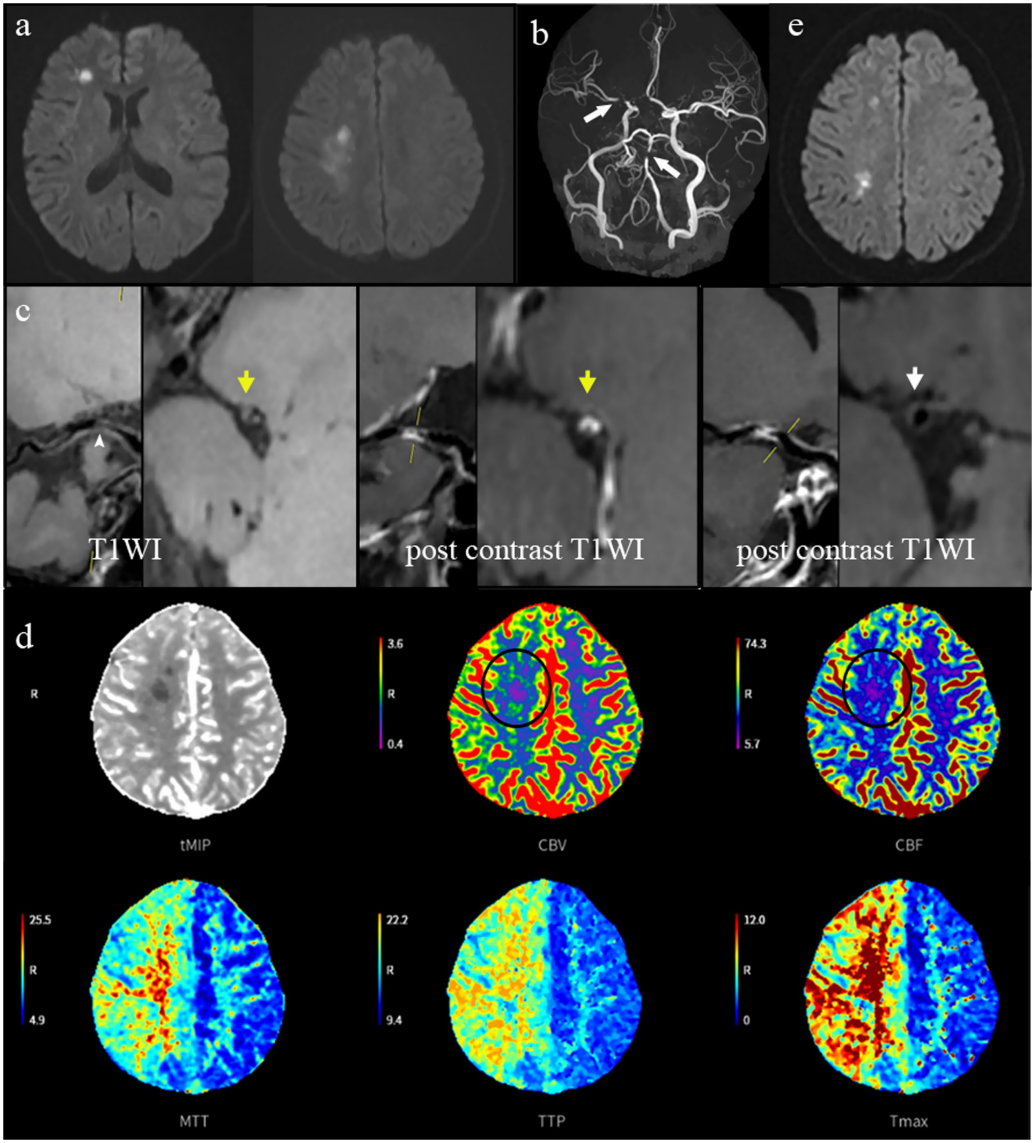
This case represents a patient with recurrent stroke. A 52-year-old male with a history of BZI experienced a recurrent stroke event 2 months after aggressive medical therapy. Baseline DWI **(a)** revealed BZI, with external (cortical) lesions located in the territories supplied by the anterior and middle cerebral arteries, as well as internal (subcortical) lesions adjacent to the lateral ventricles within the centrum semiovale. TOF-MRA **(b)** shows severe stenosis in the M1 segment and basilar artery. HRVWI **(c)** curve planes and cross-sections show the culprit lesion (yellow arrow) in the M1 segment, which was characterized by intraplaque hemorrhage (white arrowhead) on T1WI, marked contrast enhancement, and a negative remodeling pattern when compared to the reference site (white arrow). The remodel index was measured as 0.57. CTP **(d)** shows unfavorable collateral status, evidenced by reduced CBF and CBV (circle), along with prolonged MTT, TTP, and Tmax. DWI during follow-up **(e)** reveals NICLs, presenting as rose-shaped multiple inner border zone infarcts along the center of the ipsilateral centrum semiovale. BZI, border zone infarction; DWI, diffusion-weighted imaging; HRVWI, high-resolution vessel wall imaging; T1WI, T1-weighted imaging; CTP, computed tomography perfusion; CBF, cerebral blood flow; CBV, cerebral blood volume; MTT, mean transit time; TTP, time to peak; Tmax, time to maximum of the residue function; NICLs, new ischemic cerebral lesions.

During longitudinal assessment, 25 patients (32.1%) developed NICLs, with temporal analysis revealing 60% of these events occurring within the critical 3-month post-stroke period. The baseline mechanistic profile of these relapsed patients showed a predominance of BZI (14/25, 56%), followed by A-A infarcts (7/25, 9%) and POPA infarcts (4/25, 16%). A representative case is shown in Figure 2.

At the 1-year follow-up, the mechanistic distribution of NICLs showed that the BZI patterns remained most prevalent (48%, 12/25), followed by 44% A-A infarct patterns (11/25) and 8% (2/25) POPA infarct patterns.

### Recurrence risk among the three different baseline mechanistic subgroups

The three subgroups with different baseline mechanisms showed significantly different recurrence risks (*p* = 0.008). Compared with A-A infarction, baseline BZI was more likely to occur in the ERIL group (14/26 cases (53.8%) vs. 7/40 cases (17.5%), *p* = 0.002). However, there was no difference in recurrence risk between POPA and A-A infarction (*p* = 0.239) or between POPA and BZI (*p* = 0.163).

### Comparison of baseline perfusion parameters between the baseline BZI group and the non-BZI group

The baseline BZI group and non-BZI group showed no statistically significant disparities in three key perfusion parameters: CS, ischemic core volume, and mismatch area (*p* = 0.626, 0.262, and 0.464, respectively).

### Cox proportional hazards regression analysis findings

**Figure 3:**
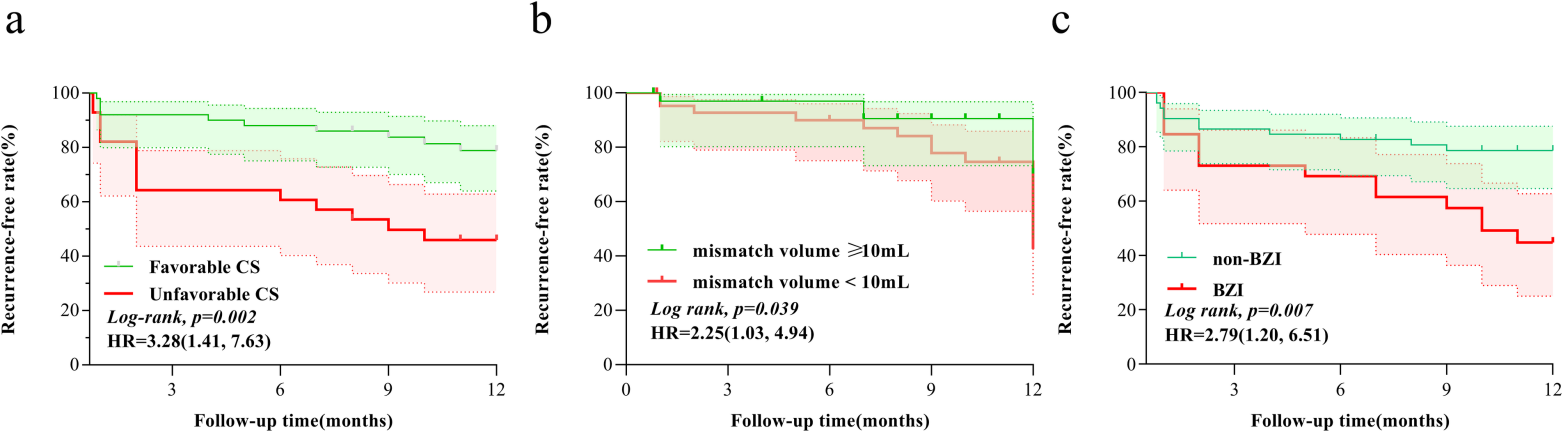
Kaplan–Meier curves of CS **(a)**, hypoperfusion mismatch volume **(b)**, and BZI **(c)** for stroke recurrence within 1 year. CS, collateral status; BZI, border zone infarcts.

**Figure 4:**
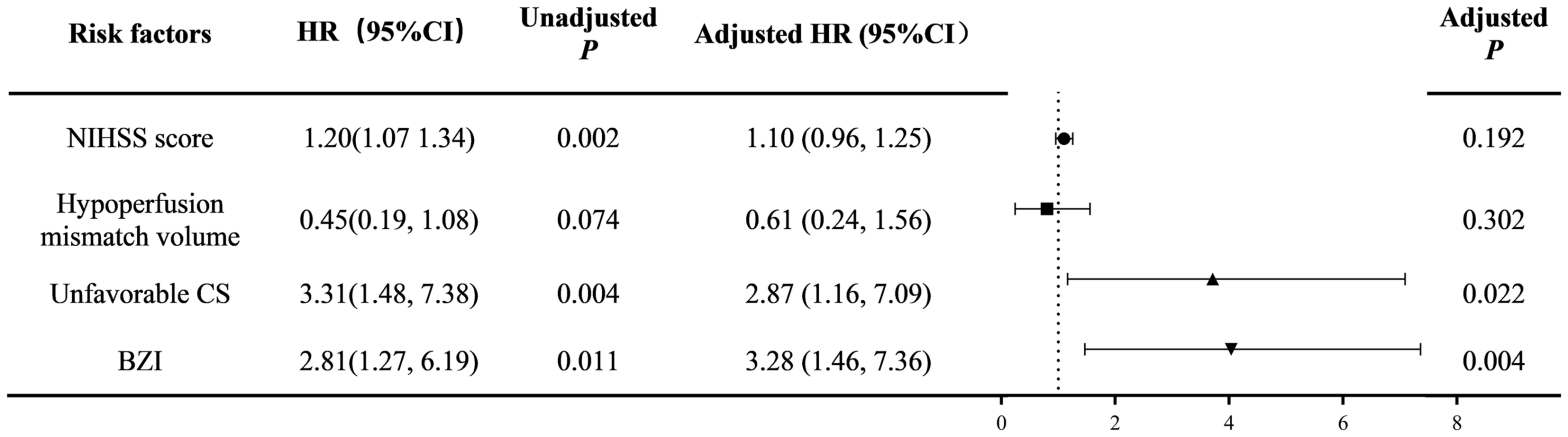
Multivariate Cox regression model of NICLs in patients with AIS during 1-year follow-up. AIS, acute ischemic stroke; NIHSS, National Institutes of Health Stroke Scale; CS, collateral status; BZI, Border zone infarcts; NICLs, new ischemic cerebral lesions.

The Kaplan–Meier survival analysis showed that CS, hypoperfusion mismatch volume, and ischemic mechanism had effects on the short-term risk of stroke recurrence (both *p* < 0.05). The multivariable Cox regression model, adjusted for key clinical and imaging parameters including cerebral perfusion characteristics (CS, hypoperfusion mismatch volume) and baseline stroke mechanism, identified baseline BZI [adjusted hazard ratio, 3.28 (95% CI, 1.49–7.36); *p* = 0.004] and CS [adjusted hazard ratio, 2.87 (95% CI, 1.16–7.09) 2.87; *p* = 0.011] as significant predictors of stroke recurrence. These results are visually presented in Figures 3, 4.

## Discussion

This study mainly investigated the characteristics of baseline plaque, perfusion, and pathogenic mechanism in patients with short-term recurrent stroke based on VWI and CTP and characterized the baseline risk factors for recurrence. Stroke recurrence occurred in 25 of the 78 patients (32%), which was greater than the 1-year recurrence rate of over 10% reported in the Stenting and Aggressive Medical Management for Preventing Recurrent Stroke in Intracranial Stenosis (SAMMPRIS) trial (1). This discrepancy may be partly explained by differences in the inclusion criteria: we excluded AIS patients who lacked either CTP or VWI imaging and who had neither, potentially introducing selection bias by restricting the cohort to a subgroup with complete imaging data, who might carry a higher inherent risk of recurrence. Additionally, recurrence in this study was radiologically defined as the presence of DWI-positive lesions, which include asymptomatic (silent) cerebral infarcts. Since such lesions are more frequently detected than clinically apparent events (30), the inclusion likely contributed to the elevated recurrence rate observed here. Our findings highlighted that the baseline perfusion status outweighed the potential risk of plaque instability in predicting ischemic recurrence. Additionally, we observed that unfavorable CS and pathogenic mechanisms held significant prognostic values and served as independent risk factors for NICLs.

Our initial focus was placed on the association between NICLs and culprit plaque features, which did not exhibit any relevance. The absence of differences in plaque characteristics between the two groups can be explained by the gradual progression of the culprit plaques. A recent prospective, 1-year follow-up longitudinal VWI study (14) found that culprit plaques exhibited an average luminal expansion of 10.9%. Additionally, the degree of plaque enhancement was observed to decrease gradually as the infarct aged; the persistence and degree of enhancement have been found to be associated with long-term stroke reoccurrence (15). Notably, other research (16) found that contrast enhancement of sICAS could persist months after the ischemic event. While positive remodeling is known to exhibit histological features of plaque vulnerability (e.g., fewer smooth muscle cells, reduced collagen content, and increased lipid accumulation) based on autopsy studies of coronary atherosclerosis (17, 18), it is thus considered a phenotype prone to rupture. In our longitudinal intracranial IVM study, there was no difference in the remodeling pattern of early recurrence. This may be influenced by the strict medical treatment, which is known to stabilize atherosclerotic plaques and reduce the risk of rupture (1), potentially masking the inherent prognostic value of vascular remodeling and contributing to the lack of observed differences in remodeling patterns between the groups.

Instead, baseline microvascular perfusion status emerged as more critical than plaque features in predicting future ischemic events—a distinction that clarifies the priority of perfusion assessment over plaque evaluation. This aligned with a previous longitudinal study (19) using arterial spin labeling and VWI. The study revealed that baseline hemodynamic impairments, instead of plaque characteristics, may be strongly associated with recurrent stroke risk and highlighted the importance of directly assessing perfusion status, which can be highly variable between patients, despite the instability of culprit plaques. Furthermore, our finding revealed significant differences in CS and penumbral volume, while no difference was observed in the volume of the ischemic core. This result suggests that insufficient CS, rather than the size of the irreversible ischemic core, is the key driver of recurrence: unfavorable CS fails to resolve the penumbra (tissue at risk of infarction), allowing this vulnerable tissue to persist and thereby increasing the likelihood of subsequent ischemic events. This finding persisted after adjusting for variables (adjusted hazard ratio 2.87, *p* = 0.011). A previous study by Saber and Liebeskind (20) found in their acute stroke cohort that baseline CS significantly influences penumbral evolution during the critical 6-h therapeutic window, highlighting its value in predicting short-term tissue outcomes. Extending prior research findings, our study reaffirms the pivotal role of CS in determining tissue outcomes beyond this 6-h therapeutic window, and this underscores the clinical prognostic relevance of robust collateral networks in medically managed AIS patients.

CS refers to the pre-existing or newly emerged vascular channels that replenish CBF when the main vascular access fails. Although the underlying mechanism of CS has not been fully clarified yet, the main approaches may include opening the existing collateral anastomosis or recruitment of the meningeal collateral that still allows for retrograde blood flow and promoting neovascularization (21). The concept of collateral perfusion can involve the arterial, tissue-level, and venous outflow levels. Emerging evidence has refined our understanding of collateral hemodynamics. A previous study (22) in AIS patients with large-vessel occlusion after thrombectomy found that the impact of CS was strongly dependent on venous vessel reperfusion. As favorable downstream venous outflow is the combined result of upstream arteries, microcirculation, and downstream drainage veins, it implies effective transit of blood through the ischemic brain tissue. Recent research (23) in medically treated AIS patients also showed that impaired intracranial venous outflow correlates with an unfavorable tissue-level collateral profile, indicates hypo-hemodynamics, and predicts a higher risk of recurrence.

Our longitudinal analysis revealed a critical temporal pattern: over half of all recurrences occurred within 3 months. More importantly, baseline mechanistic stratification demonstrated significant variations in NICL incidence across different pathological mechanism subtypes. Specifically, the baseline BZI was significantly more prevalent in the ERIL group [14 of 25 cases (56.0%), *p* = 0.001] than in the A-A group. Furthermore, the multivariable Cox regression analysis confirmed BZI as an independent predictor of recurrence (adjusted hazard ratio of 3.28, *p* = 0.004), consistent with the cohort study conducted by Kvernland et al. (24). In this study, 43.4% of symptomatic intracranial atherosclerosis (sICAS) patients developed BZI, with half of them experiencing recurrence. A comprehensive meta-analysis (25) involving 1,219 AIS patients, including 341 with BZI, further substantiated this association, showing that BZI had a twofold risk of adverse prognosis—neurological deterioration or recurrent stroke—and a nearly threefold increased risk in those with anterior circulation involvement compared to patients without BZI. Collectively, these findings suggest that BZI serves as an independent biomarker for early stroke recurrence, particularly within the 90-day vulnerable period.

However, the majority of prior studies have focused on the progression of neurodegeneration, which encompasses a heterogeneous range of pathophysiological mechanisms including stroke recurrence, cerebral edema, and hemorrhagic transformation, failing to elucidate the relationship between neurodegeneration and recurrence. Therefore, these studies may introduce subjective bias in outcome evaluation. In contrast, our study defined recurrence as DWI-confirmed NICLs within the same vascular territory. The new BZI occurred in 12 of 25 NICLs, among which 3 even presented as bilateral NICLs. It might reflect underlying hemodynamic impairment and further reinforce the hemodynamic vulnerability hypothesis of recurrent cerebral infarction in patients with sICAS (26).

When considering the baseline CS between the BZI and non-BZI groups, which provides essential compensatory blood supply to tissue after ischemic injury, there was no significant difference. This lack of difference may be attributed to the distinct collateral supply mechanisms in the two types of BZI. The internal BZI, located in the lenticulostriate-MCA border zone, is supplied by the terminal branches of deep perforating lenticulostriate arteries and has little collateral supply. In contrast, the external BZI is situated in the cortical border zones between the middle and anterior or middle and posterior cerebral arteries, adjacent to the origin of the pial-perforating arteries. As a result, it has a greater potential for collateral supply through leptomeningeal or dural anastomoses (27).

Beyond anatomical collateral differences, internal BZI was more frequently associated with anterograde blood flow impairment in MCA-M1 lesions, assessed via CTA-based computational fluid dynamics (28), and higher plaque burden, evaluated via VWI (29), was more frequently observed in internal BZI compared to external BZI. Conversely, external BZI was more likely concomitant with small cortical infarcts (28). Therefore, external BZI is considered to be primarily embolic in nature, with insufficient perfusion hindering the clearance (washout) of emboli in cortical border zones, where the perfusion is lower than that in other vascular territories. Recently, Li et al. (28) further verified that medically treated patients with isolated internal BZI had a significantly higher risk of recurrence within the first 3 months than those with isolated external BZI. They also noted that current pharmacological treatments for early stroke prevention are effective in cortical BZI through plaque stabilization. However, for internal BZI—where hypoperfusion serves as the dominant pathological mechanism underlying stroke—this conventional pharmacotherapy fails to provide adequate prevention and may even be clinically unsuitable.

In summary, BZI and unfavorable CS profoundly reflect the pathological state of exhausted cerebral hemodynamic reserve. This study confirms that the hemodynamic impairment represented by BZI and unfavorable CS serves as a core driver of early stroke recurrence. This finding carries major clinical implications: it suggests that for such patients, conventional medical therapy—which primarily targets embolic and thrombotic pathways—has reached its limits and cannot fundamentally reverse the state of hypoperfusion.

## Limitations

First, due to the single-center nature of the study, the small cohort was insufficient for multivariate analyses. Consequently, we incorporated some mixed mechanisms into a single stroke mechanism subgroup, largely based on the main distribution characteristics of the lesions, in statistical analyses for the associations between stroke mechanisms and outcomes. Specifically, among patients with mixed infarct patterns, those with perforator territory distribution were classified as the major POPA subgroup. The remaining patients who had a border zone distribution infarct as part of their mixed infarct patterns were categorized into the BZI group. It could take into account possible confounding factors. Given the small cohort, we pooled two different types of ischemic infarctions, intracortical (subcortical) and extracortical (cortical), into one group, which have different pathogeneses and prognoses. In this exploratory study, our results revealed the association between pathological mechanisms and recurrence; however, the limitations associated with sample size may constrain statistical robustness, increase the risk of overfitting, and necessitate validation in larger, multicenter cohorts.

Second, patient selection may be biased when retrospective analysis of prospectively collected data from cohort studies is performed. For instance, due to the lack of detailed symptom records and negative imaging examinations, we excluded some patients with TIA, and these patients were in the early stage of pathological changes of cerebral ischemic infarction and with high recurrence risk. Additionally, the retrospective study design limits access to detailed longitudinal data on medical management, including patient adherence to antiplatelet or statin therapy and subsequent dose adjustments. As a result, the influence of these unmeasured confounders on observed recurrence rates cannot be fully ruled out.

Third, for some patients with small or scattered lesions on DWI, the ischemic core on CTP may be underestimated, especially for BZI, which is mostly manifested as scattered distribution. Moreover, based on the perfusion parameters (particular rCBF), we categorized collateral circulation into two types based on indirect classification: unfavorable(misery perfusion), characterized by a reduction in rCBF, and favorable perfusion, associated with normal rCBF. However, it does not capture the dynamic changes in the flow state within vessels. Alberta Stroke Program Early CT Score and Digital Subtraction Angiography, in combination with the filling status and filling time delay of collateral angiography agents, can better evaluate the collateral circulation status (20). Furthermore, we limited the results to NICLs, including asymptomatic (silent) infarction; our findings may not be directly comparable to studies that define recurrence based on clinical events, potentially affecting the generalizability of the conclusions.

## Conclusion

In the medical management of AIS, greater emphasis should be placed on the perfusion pathophysiology underlying the ischemic process when assessing the prognostic value for stroke recurrence—rather than focusing solely on the atherosclerotic plaque itself. Additionally, BZI and unfavorable CS were identified as independent factors associated with an elevated risk of recurrent stroke. Consequently, it is essential to optimize individualized therapeutic strategies and enhance secondary prevention efforts to mitigate the risk of short-term stroke recurrence.

## Data Availability

The original contributions presented in the study are included in the article/Supplementary material, further inquiries can be directed to the corresponding author.

## Glossary

AIS: Acute ischemic stroke
sICAS: Symptomatic intracranial atherosclerotic stenosis
NICLs: New ischemic cerebral lesions
ERILs: Early recurrence ischemic lesions
MCA: Middle cerebral artery
VWI: Vessel wall imaging
HR-VWI: High-resolution vessel wall imaging
CTP: Computed tomography perfusion
IPH: Intraplaque hemorrhage
CS: Collateral status
A-A: Artery-to-artery embolism
BZI: Border, zone infarction
POPA: Parent artery occlusion of penetrating arteries
NIHSS: National Institutes of Health Stroke Scale
CBF: Cerebral blood flow
CBV: Cerebral blood volume
Tmax: Tissue with delayed time to maximum
DWI: Diffusion-weighted imaging
MRA: Magnetic resonance angiography
TIA: Transient ischemic attack
SAMMPRIS: Stenting and Aggressive Medical Management for Preventing Recurrent Stroke in Intracranial Stenosis
