# Supplementary material for: Factors associated with short-term recurrent ischemic stroke: culprit plaque, collateral circulation, and pathological mechanisms
Source: Front Neurol. 2025 Dec 18;16:1720513. doi: 10.3389/fneur.2025.1720513 (PMC12756096; doi:10.3389/fneur.2025.1720513)
Supplement: Supplementary file 1 [file Table_1.docx]

SUPPLEMENTAL MATERIAL

Table 1. Parameters of HR-VWI MRI sequences

| MR scan parametes | 3D T1WI CUBE Pre &Postcontrast | 3D T2WI CUBE | MRA |
| --- | --- | --- | --- |
| TR/TE, ms | 650/12 | 2502/118 | 3.4/18 |
| voxel size, mm^3^ | 0.8×0.8×0.8 | 0.8×0.8×0.8 | 0.7×0.9×1.2 |
| Slice thickness (mm) | 0.8 | 0.8 | 1.2 |
| Echo train length | 36 | 96 | 1 |
| Flip angle (degrees) | Variable Flip angle flag | Variable Flip angle flag | 20 |
| Field of view(mm^3^) | 192×192 | 192×192 | 220×206 |
| Matrix(frequency×phase) | 240×240 | 240×240 | 320×256 |
| Pixel Bandwidth | 244 | 244 | 162 |
| Intensity correction | SCENIC | SCENIC | PURE |
| Acquisition time(s) | 369 | 274 | 194 |

HR-VWI, high-resolution vessel wall imaging; TR, repetition time; TE echo time.
